# Uso de curativo a vácuo como terapia adjuvante na cicatrização de sítio cirúrgico infectado

**DOI:** 10.1590/1677-5449.002816

**Published:** 2016

**Authors:** Paula Angeleli Bueno de Camargo, Matheus Bertanha, Regina Moura, Rodrigo Gibin Jaldin, Ricardo de Alvarenga Yoshida, Rafael Elias Farres Pimenta, Jamil Victor de Oliveira Mariúba, Marcone Lima Sobreira

**Affiliations:** 1 Universidade Estadual Paulista “Júlio de Mesquita Filho” – UNESP, Faculdade de Medicina de Botucatu, Departamento de Cirurgia e Ortopedia, São Paulo, SP, Brasil.

**Keywords:** terapêutica, cicatrização, técnicas de fechamento de ferimentos abdominais, infecção, prótese vascular

## Abstract

Infecções de sítios cirúrgicos com envolvimento de próteses sintéticas constituem grande desafio para tratamento. Apresentamos o caso de uma paciente com múltiplas comorbidades, histórico de enxerto aortobifemoral há 6 anos e reabordagem das anastomoses femorais por reestenoses há 5 anos. Apresentou dor inguinal esquerda e abaulamento súbitos com diagnóstico de pseudoaneurisma femoral roto e instabilidade hemodinâmica. Foi submetida a correção emergencial com interposição de prótese de dácron recoberta por prata e correção de grande hérnia incisional abdominal com tela sintética ao mesmo tempo. No pós-operatório, manteve-se por longo período sob terapia intensiva com dificuldade de extubação. Nesse ínterim, apresentou deiscência das suturas e fístula purulenta inguinal esquerda em contato com a prótese vascular. Optou-se pelo tratamento conservador, com desbridamento das feridas e aplicação de curativo a vácuo. A paciente evoluiu com melhora e cicatrização das feridas. Essa pode se constituir em ferramenta importante em casos similares.

## INTRODUÇÃO

As deiscências de incisões cirúrgicas, particularmente com envolvimento de próteses sintéticas, constituem grande desafio para tratamento^1^. Nas próteses vasculares, em particular, há um microambiente favorável à produção de biofilme, que sustenta a colonização bacteriana e encapsula os germes, protegendo-os contra as defesas naturais do organismo e da terapia antibiótica^1^. O tratamento geralmente preconizado é a remoção da prótese infectada^1^. Porém, essas cirurgias são geralmente maiores que as prévias, uma vez que apresentam forte envolvimento inflamatório. Assim, o paciente deve ter boas condições clínicas para suportar uma cirurgia que pode envolver reconstruções vasculares extra-anatômicas complexas, que demandam maior tempo operatório e elevam as taxas de morbimortalidade. Dessa forma, pretende-se evitar a progressão da infecção, quadros isquêmicos graves decorrentes da remoção isolada da prótese e o risco de amputações. Nesse contexto, apresentamos um caso de sucessivas complicações cirúrgicas decorrentes de reintervenções emergenciais sobre um enxerto aortobifemoral antigo, realizado há 6 anos para o tratamento de isquemia crítica de membros inferiores. Retornou com degeneração da artéria femoral comum esquerda e constituiu um grande desafio terapêutico.

## PARTE I - SITUAÇÃO CLÍNICA

Paciente do sexo feminino, branca, com 75 anos de idade, dislipidêmica, tabagista ativa com doença pulmonar obstrutiva crônica, doença renal crônica, obesidade mórbida e portadora de insuficiência cardíaca. Tinha histórico de cirurgia convencional de enxerto aortobifemoral com prótese de dácron bifurcada, realizada para o tratamento de isquemia crítica de membros inferiores 6 anos atrás, quando havia uma situação clínica mais favorável. O quadro de isquemia crítica inicial caracterizava-se por claudicação intermitente, lesões tróficas em ambos os pés (necrose puntiforme de pododáctilos) e dor em repouso. Como complicação daquela cirurgia, no pós-operatório, teve deiscência da incisão abdominal com consequente hérnia incisional.

Um ano após a primeira cirurgia, a paciente apresentou necrose em região calcânea esquerda, que foi relacionada a uma piora da perfusão desse membro. Isso foi confirmado por meio de ultrassom dúplex colorido, o qual demonstrou estenose > 75% nas anastomoses femorais. A correção foi realizada com reabordagem cirúrgica das anastomoses e confecção de remendo de dácron, com sucesso técnico. A evolução pós-operatória trouxe compensação circulatória para os membros inferiores e cicatrização da ferida.

Após 5 anos de acompanhamento ambulatorial regular, deu entrada no pronto-socorro de nossa instituição queixando-se de dor forte e abaulamento súbito em região inguinal esquerda. Clinicamente, a paciente apresentou instabilidade hemodinâmica e, após um rastreamento emergencial com ultrassom dúplex colorido, visualizou-se pseudoaneurisma na interface entre a artéria femoral e o remendo de dácron e extravasamento de sangue para o retroperitônio. Foi indicado tratamento cirúrgico de emergência.

Foi, então, realizada correção cirúrgica convencional, com remoção do remendo e interposição de segmento de dácron recoberto por prata entre a porção média do ramo esquerdo da prótese aortobifemoral e a bifurcação femoral. Além disso, a hérnia incisional abdominal prévia foi corrigida por meio de colocação de tela sintética no mesmo ato cirúrgico, pela equipe de gastrocirurgia.

Em decorrência das várias comorbidades, a evolução pós-operatória foi desfavorável, permanecendo a paciente em regime de terapia intensiva por longo período. Ocorreu deiscência de sutura das incisões inguinal e abdominal com drenagem de secreção purulenta na inguinotomia esquerda. Detectou-se, por tomografia computadorizada, uma fístula de drenagem da coleção adjacente ao enxerto pela inguinotomia, sem exposição direta da prótese. Foi realizada a cultura das secreções, que se apresentou positiva para *Staphylococcus epidermidis* e *Staphylococcus aureus* coagulase negativo, ambos sensíveis à vancomicina. A situação clínica da paciente era agravada, ainda, por quadro de pneumonia, tratada com imipenem, tazocin e polimixina B, e pelo antecedente de doença pulmonar obstrutiva crônica, o que a manteve em ventilação mecânica invasiva por 45 dias.

Diante desse quadro e sem condições cirúrgicas, as opções de tratamento eram:

- Antibióticos sistêmicos, desbridamentos e curativos locais;
- Lavagem contínua dos sítios infectados com antissépticos e antibióticos;
- Remoção cirúrgica de todas as próteses, apesar das condições clínicas desfavoráveis, seguida de reconstrução vascular extra-anatômica;
- Remoção cirúrgica de todas as próteses, apesar das condições clínicas desfavoráveis, e espera por delimitação isquêmica para posterior amputação;
- Curativo a vácuo, antibioticoterapia e observação das condições clínicas da paciente.

## PARTE II – O QUE FOI FEITO

Foi instalado curativo a vácuo nas deiscências do abdome e região inguinal esquerda. O equipamento usado foi o Sistema de Terapia V.A.C. ATS® (KCI Kinetic Concepts Inc, San Antonio, Texas, EUA) e antibioticoterapia (vancomicina e imipenem por 21 dias) com observação das condições clínicas da paciente.

**Figura 1:**
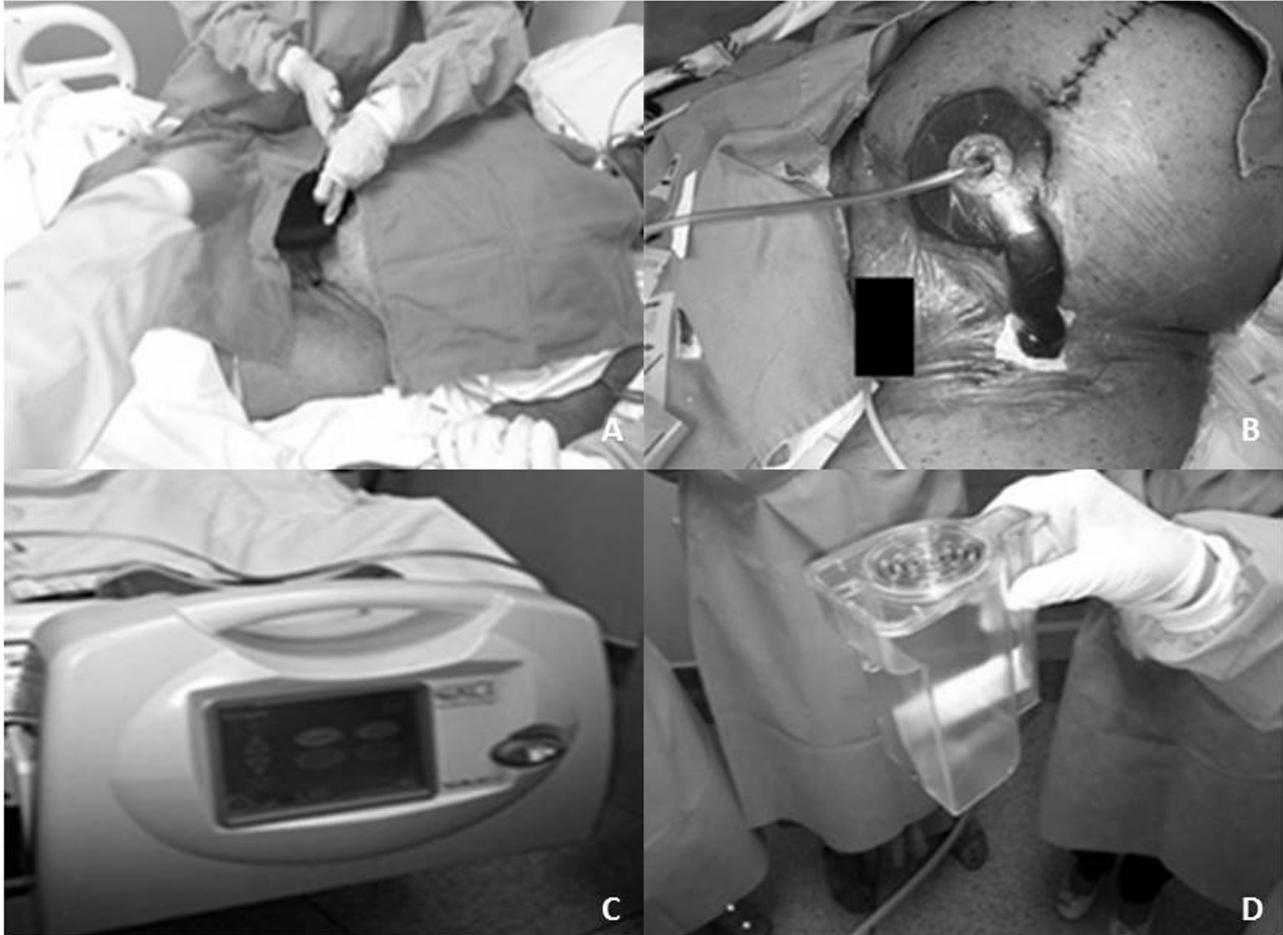
Aplicação do curativo a vácuo após o desbridamento das incisões deiscentes. (A) Troca do curativo com a colocação da esponja de poliuretano no leito das feridas; (B) Kit de sucção e filme plástico devidamente instalados; (C) Equipamento de pressão negativa fixado no leito da paciente; (D) Refil do frasco de drenagem do curativo a vácuo.

**Figura 2:**
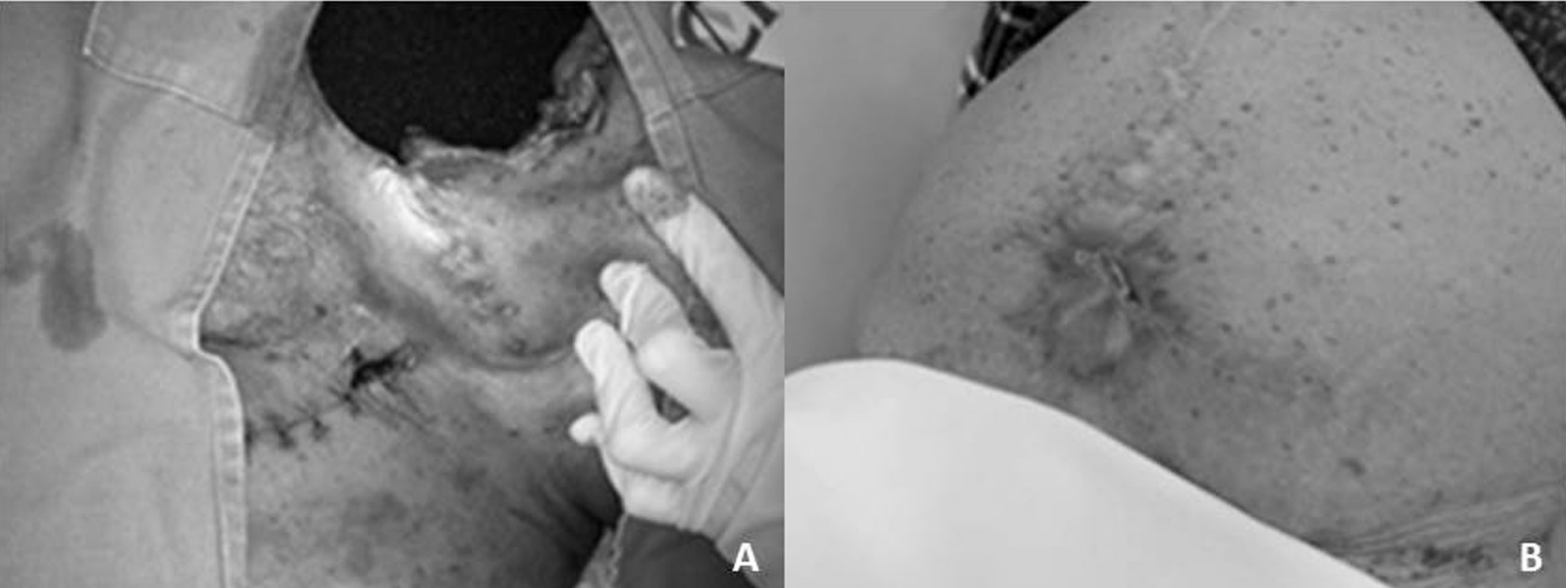
Processo de cicatrização das feridas com uso do curativo a vácuo. (A) Resultado intermediário do processo de cicatrização, ainda em uso de curativo a vácuo; (B) Resultado final do processo de cicatrização das feridas.

Após desbridamento das feridas, foi colocada esponja de poliuretano exclusivamente dentro do leito das deiscências, com cobertura total com filme plástico (Figuras 1A e 1B). O tubo de sucção foi acoplado ao equipamento de pressão negativa, mantendo-se uma pressão de -125 mmHg de forma contínua (Figuras 1C e 1D). A troca dos kits de sucção foi feita a cada 3 dias. Houve drenagem de, aproximadamente, 50 mL/dia de secreção purulenta. A paciente evoluiu com estabilização das comorbidades e controle da infecção, extubação após 45 dias, fechamento progressivo das deiscências incisionais, granulação total em 60 dias com suspensão do uso do curativo a vácuo, curativos convencionais com hidrogel durante internação por mais 25 dias e fechamento ambulatorial das feridas após 9 meses de tratamento (Figuras 2A e 2B).

## DISCUSSÃO

O uso de curativos com pressão negativa para tratamentos diversos é conhecido desde a antiguidade^2^. O tratamento de feridas crônicas com terapia a vácuo padronizada teve início em 1997^3^. Sua ação é baseada nos conceitos de: contração da ferida, eliminação do exsudado e do tecido inviável, estímulo à mitose celular, manutenção de um ambiente úmido, redução do edema tecidual, remoção de bactérias, melhora da vascularização e aceleração da granulação^4^^-^ 7.

Pode-se considerar a indicação de tratamento com curativo a vácuo para feridas com baixa resposta ao tratamento convencional, em que se prevê um longo período para cicatrização, feridas profundas e com elevada quantidade de exsudado e como coadjuvante a outras terapias ou intervenções. As contraindicações são: feridas com malignidade, fístulas para órgãos e cavidades, osteomielites e exposição de vasos sanguíneos com risco de sangramento.

Pode-se, ainda, atribuir como vantagens dessa terapia a redução da inflamação e da dor resultantes da manipulação constante da ferida, isenção de contaminação por contato e conforto para o paciente, uma vez que não deixa odores desagradáveis. Em contrapartida, apresenta custos imediatos mais elevados, principalmente relacionados às trocas do refil e do próprio curativo em condições assépticas ao menos uma vez por semana. Entretanto, quando se somam todos os benefícios do uso do curativo a vácuo em comparação com os curativos convencionais, fica evidente que há uma relação de custo-efetividade com a adoção do curativo a vácuo. Com relação ao caso previamente apresentado, provavelmente um curativo convencional teria dificuldade em manter um ambiente propício à cicatrização, levando-se em consideração a presença de uma fístula purulenta em contato com a prótese arterial implantada e as grandes áreas deiscentes.

Revisões sistemáticas^8^^,^ 9 e um estudo randomizado^10^ mostraram a efetividade do curativo com pressão negativa em várias situações, com relação à proporção de feridas cicatrizadas e velocidade de fechamento das mesmas, sendo particularmente efetivo em pés diabéticos^11^^-^ 13, enxertos de pele^14^ e infecções pós-cirúrgicas^15^^,^ 16. No entanto, faltam ainda estudos randomizados de boa qualidade e isentos de conflitos de interesse para melhor avaliar esse método^17^.

As complicações descritas como relacionadas ao uso do curativo a vácuo são infrequentes, sendo a maioria relacionada a dor local, hipertrofia do tecido de granulação e danos aos vasos sanguíneos adjacentes^18^^,^ 19. Ressalta-se, então, que a esponja do curativo a vácuo não deve ser aplicada diretamente sobre vasos sanguíneos. Nessa situação, um filme de silicone não adesivo deve servir de anteparo como proteção de interface entre a esponja e o tecido, evitando a erosão do vaso^15^.

A pressão negativa nas feridas é geralmente usada de forma contínua, mas existem equipamentos que podem atuar de forma intermitente ou variável, não sendo possível encontrar evidências clínicas de vantagem relacionada a essa variável^19^. Níveis de pressão negativa menores que 80 mmHg (pressão negativa) são recomendados para se obter algum efeito do tratamento^20^. A instilação de fluidos no leito da ferida pode melhorar a eficiência do tratamento em alguns casos^21^.

Pode-se concluir que os curativos com pressão negativa têm recomendações bem estabelecidas para o tratamento de feridas com características variadas, sendo que podem apresentar redução no tempo de cicatrização de feridas, maior conforto para o paciente e raras complicações. No caso apresentado, para o qual houve aprovação do Comitê de Ética de nossa instituição e que não apresenta conflito de interesse, o curativo a vácuo foi uma importante ferramenta para o sucesso terapêutico em uma situação de exceção, na qual a abordagem cirúrgica para remoção das próteses arteriais estaria associada a um elevado risco cirúrgico e de amputação. A conduta adotada apresentou um resultado bastante satisfatório.
