# Supplementary material for: Uso de curativo a vácuo como terapia adjuvante na cicatrização de sítio cirúrgico infectado
Source: J Vasc Bras. 2016 Oct-Dec;15(4):312–6. [Article in Portuguese] doi: 10.1590/1677-5449.002816 (PMC5829731; doi:10.1590/1677-5449.002816)
Supplement: Translation

# Translation: Using vacuum therapy as an adjunctive treatment for healing of infected surgical sites

**Authors:** Paula Angeleli Bueno de Camargo, Matheus Bertanha, Regina Moura, Rodrigo Gibin Jaldin, Ricardo de Alvarenga Yoshida, Rafael Elias Farres Pimenta, Jamil Victor de Oliveira Mariúba, Marcone Lima Sobreira

**Affiliations:** 1 Universidade Estadual Paulista “Júlio de Mesquita Filho” – UNESP, Faculdade de Medicina de Botucatu, Departamento de Cirurgia e Ortopedia, São Paulo, SP, Brazil.

**Keywords:** treatment, healing, abdominal injury closure techniques, infection, vascular prosthesis

## Abstract

Infections at the sites of surgery involving synthetic prostheses are challenging to treat. We present a case of a patient with multiple comorbidities who had undergone an aortobifemoral bypass 6 years previously and then re-intervention at the femoral anastomoses for restenosis 5 years previously. The patient presented with acute left inguinal pain and swelling and was diagnosed with a ruptured femoral pseudoaneurysm and hemodynamic instability. A repair was conducted by interposition of a silver-coated Dacron graft in the emergency room, and a large abdominal incisional hernia was repaired with synthetic mesh during the same intervention. After surgery, the patient remained intubated in intensive care for a long period. Meanwhile, she presented dehiscence of sutures and a left inguinal purulent fistula that was in contact with the vascular prosthesis. Conservative treatment was chosen, with debridement of wounds and vacuum therapy. The patient improved and the wounds healed. This could be an important tool in similar cases.

## INTRODUCTION

Dehiscence of surgical incisions is a major challenge to treat, particularly when they involve synthetic prostheses.^1^ Vascular prostheses in particular provide a microenvironment that is conducive to production of biofilm, which supports bacterial colonization and encapsulates the germs, protecting them from the body’s natural defenses and from antibiotics.^1^ The treatment that is generally recommended is removal of the infected prosthesis.^1^ However, these operations are generally of a larger scale than the original surgery because of the significant inflammatory involvement. The patient must therefore be in good clinical conditions to withstand a surgical operation that may include complex extra-anatomic vascular reconstruction, requiring longer operating times and elevating morbidity and mortality rates. The objectives are to prevent continued development of the infection, to avoid severe ischemia resulting from simply removing the prosthesis, and to reduce the risk of amputations. Against this background, we present a case of successive surgical complications triggered by emergency reinterventions to repair a previous aortobifemoral bypass that had been constructed 6 years previously to treat critical lower limb ischemia. The patient presented with degeneration of the left common femoral artery and the case was a significant therapeutic challenge.

## PART I – CLINICAL SITUATION

The patient was a white, 75-year-old, female, active smoker with dyslipidemia, chronic obstructive pulmonary disease, chronic kidney disease, morbid obesity, and heart failure. Six years previously she had undergone conventional surgery to construct an aortobifemoral bypass using a bifurcated dacron graft to treat critical lower limb ischemia, when her clinical situation had been less unfavorable. Her initial critical ischemia had presented with intermittent claudication, trophic ulcers on both feet (necrosis punctiform of the toes), and pain at rest. The patient had already suffered a complication during the postoperative period of that operation: dehiscence of the abdominal incision, causing an incisional hernia.

One year after the first operation, the patient presented once more, with necrosis of the left heel, which was related to deterioration of perfusion to the left lower limb. This was confirmed with color duplex ultrasound, which showed > 75% stenosis of the femoral anastomoses. A surgical reintervention was conducted to repair the anastomoses with a dacron patch and was a technical success. Postoperative recovery was accompanied by compensation of the lower limb circulation and the wound healed.

After 5 years of regular outpatients follow-up, the patient was admitted to the emergency room at our institution complaining of severe pain and swelling in the left inguinal region. Clinically, the patient was hemodynamically unstable and emergency duplex ultrasound screening revealed a pseudoaneurysm at the interface between the femoral artery and the dacron patch, with blood leaking into the retroperitoneal space. The patient was prepared for emergency surgical treatment.

The pseudoaneurysm was repaired during a conventional surgical operation, with removal of the patch and interposition of a silver-coated dacron segment between the medial portion of the left branch of the aortobifemoral graft and the femoral bifurcation. During the same operation, the gastric surgery team repaired the preexisting incisional hernia by placement of a synthetic mesh.

The multiple comorbidities caused unfavorable postoperative progress and the patient was kept in intensive care for a long period of time. The inguinal and abdominal sutures underwent dehiscence and there were purulent secretions draining from the left inguinotomy. On computed tomography, a fistula was detected draining pus adjacent to the graft via the inguinotomy, to which the prosthesis was not directly exposed. The secretions were cultured and found to be positive for *Staphylococcus epidermidis* and coagulase negative *Staphylococcus aureus*, both sensitive to vancomycin. The patient’s clinical situation was further aggravated by pneumonia, which was treated with imipenem, tazocin and polymyxin B, and by the preexisting chronic obstructive pulmonary disease, which kept her on invasive mechanical ventilation for 45 days.

Faced with this clinical status and without the necessary conditions for surgery, the treatment options were as follows:

- Systemic antibiotics, debridement and local dressings;
- Continuous cleaning of the infected sites with antiseptics and antibiotics;
- Surgical removal of all prostheses, despite the unfavorable clinical conditions, followed by extra-anatomic vascular reconstruction;
- Surgical removal of all prostheses, despite the unfavorable clinical conditions, and amputation after delimitation of ischemia;
- Vacuum dressing, antibiotic therapy and observation of the patient’s clinical condition.

## PART II – WHAT WAS DONE

A vacuum dressing was applied to the dehiscences at the abdomen and left inguinal region. The V.A.C. ATS Therapy System® (KCI Kinetic Concepts Inc, San Antonio, Texas, United States) was used in combination with antibiotic therapy (vancomycin and imipenem for 21 days) and observation of the patient’s clinical conditions.

After debridement of the wounds, polyurethane foam was placed exclusively within the dehiscence bed and completely covered with plastic film (Figures 1A and 1B). The suction tube was fitted to the negative pressure unit, maintaining a pressure of -125 mmHg continuously (Figures 1C and 1D). Suction kits were changed every 3 days. Approximately 50 mL/day of purulent secretions were drained. The patient’s comorbidities stabilized and the infection was controlled. She was extubated after 45 days, and the incisional dehiscences closed progressively, achieving total granulation at 60 days, when the vacuum dressing was withdrawn, followed by conventional hydrogel dressings for a further 25 days while still in hospital, and the wounds closed during outpatients follow-up 9 months after starting treatment (Figures 2A and 2B).

**Figure 1:** Application of vacuum dressing after debridement of dehiscent incisions. (A) Dressing change, placing polyurethane foam on wound beds; (B) Suction kit and plastic film correctly fitted; (C) Negative pressure unit attched to patient’s bed; (D) Replacement vacuum dressing drainage jar.

**Figure 2:** Wound healing process with vacuum dressing. (A) Intermediate stage of healing process, vacuum dressing still in use; (B) Final result of wound healing process.

## DISCUSSION

Utilization of dressings with negative pressure has been known since ancient times for a range of treatments.^2^ Treatment of chronic wounds using standardized vacuums began in 1997.^3^ The effects are based on the following concepts: contraction of the wound, removal of exudate and non-viable tissues, stimulation of cell mitosis, maintenance of a humid environment, reduction of tissue edema, removal of bacteria, improvement of vascularization, and acceleration of granulation.^4^^-^ 7

Treatment with vacuum dressings may be indicated for wounds that do not respond well to conventional treatment, when a long healing period is predicted, for deep wounds and those with high quantities of exudate, and as a supplementary technique in combination with other treatments or interventions. Contraindications are: wounds with malignancies, fistulae into organs and cavities, osteomyelitis, and exposure of blood vessels at risk of bleeding.

Advantages of this treatment include reduction of inflammation and pain caused by constant manipulation of the wound, exclusion of contamination by contact, and improved patient comfort, since it does not produce unpleasant odors. On the other hand, immediate costs are high, primarily related to changing the refill and the dressing itself under aseptic conditions at least once a week. However, when all the benefits of using a vacuum dressing compared with conventional dressings are added up, it is clear that the choice of a vacuum dressing offers a good cost-effectiveness ratio. With regard to the case described above, it is probable that conventional dressings would have been unlikely to have successfully maintained an environment conducive to healing, considering the presence of a purulent fistula in contact with the prosthetic arterial graft and the large areas of dehiscence.

Systematic reviews^8^^,^ 9 and a randomized study^10^ show the effectiveness of negative pressure dressings in a range of situations, in terms both of the proportion of wounds healed and the speed with which they close, and they are particularly effective for diabetic feet,^11^^-^ 13 skin grafts^14^ and infections after surgery.^15^^,^ 16 However, there is still a lack of good quality randomized studies free from conflicts of interest that would allow the method to be evaluated more thoroughly.^17^

Complications that have been described in relation to vacuum dressings are uncommon and the majority are related to local pain, hypertrophy of granulation tissue, and damage to adjacent blood vessels.^18^^,^ 19 It should however be stressed that the dressing foam should not be placed in direct contact with blood vessels. In such situations, a non-adhesive silicone film should be used as a pre-preparation as a protection for the interface between foam and tissue, preventing erosion of the vessel.^15^

In general the negative pressure is applied to wounds continuously, but there are systems that can provide intermittent or variable action, although there is no clinical evidence that this variable offers advantages.^19^ Negative pressure levels below 80 mmHg (negative pressure) are recommended to obtain treatment effectiveness.^20^ In some cases instillation of fluids to the wound bed can improve efficacy.^21^

It can be concluded that there are well-established recommendations for using negative pressure dressing to treat wounds with a variety of characteristics and they can offer reductions in the time taken for wounds to heal, combined with greater patient comfort and rare complications. In the case described here, which was approved by our institutional Ethics Committee and does not involve any conflicts of interests, the vacuum dressing was an important tool for achieving therapeutic success in an exceptional situation, in which an additional surgical operation to remove the arterial prostheses would have incurred a high surgical risk and high risk of amputation. The conduct chosen achieved very satisfactory results.
